# Evaluating pooled testing for asymptomatic screening of healthcare workers in hospitals

**DOI:** 10.1186/s12879-023-08881-x

**Published:** 2023-12-21

**Authors:** Bethany Heath, Stephanie Evans, David S. Robertson, Julie V. Robotham, Sofía S. Villar, Anne M. Presanis

**Affiliations:** 1 MRC Biostatistics Unit, Univeristy of Cambridge, Robinson Way, Cambridge, CB2 0SR Cambridgeshire United Kingdom; 2 HCAI, Fungal, AMR, AMU and Sepsis Division, UK Health Security Agency, London, United Kingdom; 3 Statistics, Modelling and Economics Division, UK Health Security Agency, London, United Kingdom; 4 NIHR Health Protection Research Unit in Modelling and Health Economics at Imperial College London in partnership with the UK Health Security Agency and London School of Hygiene and Tropical Medicine, London, United Kingdom; 5 NIHR Health Protection Research Unit in Healthcare Associated Infections and Antimicrobial Resistance at University of Oxford in partnership with the UK Health Security Agency, Oxford, United Kingdom; 6 NIHR Health Protection Research Unit in Behavioural Science and Evaluation at University of Bristol in partnership with the UK Health Security Agency and MRC Biostatistics Unit, University of Cambridge, Bristol, United Kingdom

**Keywords:** Nosocomial transmission, Pandemic preparedness, Simulation study, Testing policy

## Abstract

**Background:**

There is evidence that during the COVID pandemic, a number of patient and HCW infections were nosocomial. Various measures were put in place to try to reduce these infections including developing asymptomatic PCR (polymerase chain reaction) testing schemes for healthcare workers. Regularly testing all healthcare workers requires many tests while reducing this number by only testing some healthcare workers can result in undetected cases. An efficient way to test as many individuals as possible with a limited testing capacity is to consider pooling multiple samples to be analysed with a single test (known as pooled testing).

**Methods:**

Two different pooled testing schemes for the asymptomatic testing are evaluated using an individual-based model representing the transmission of SARS-CoV-2 in a ‘typical’ English hospital. We adapt the modelling to reflect two scenarios: a) a retrospective look at earlier SARS-CoV-2 variants under lockdown or social restrictions, and b) transitioning back to ‘normal life’ without lockdown and with the omicron variant. The two pooled testing schemes analysed differ in the population that is eligible for testing. In the ‘ward’ testing scheme only healthcare workers who work on a single ward are eligible and in the ‘full’ testing scheme all healthcare workers are eligible including those that move across wards. Both pooled schemes are compared against the baseline scheme which tests only symptomatic healthcare workers.

**Results:**

Including a pooled asymptomatic testing scheme is found to have a modest (albeit statistically significant) effect, reducing the total number of nosocomial healthcare worker infections by about 2$\%$ in both the lockdown and non-lockdown setting. However, this reduction must be balanced with the increase in cost and healthcare worker isolations. Both ward and full testing reduce HCW infections similarly but the cost for ward testing is much less. We also consider the use of lateral flow devices (LFDs) for follow-up testing. Considering LFDs reduces cost and time but LFDs have a different error profile to PCR tests.

**Conclusions:**

Whether a *PCR-only* or *PCR and LFD ward* testing scheme is chosen depends on the metrics of most interest to policy makers, the virus prevalence and whether there is a lockdown.

**Supplementary Information:**

The online version contains supplementary material available at 10.1186/s12879-023-08881-x.

## Background

Nosocomial SARS-CoV-2 infections in both patients and healthcare workers (HCWs) have been documented throughout the SARS-CoV-2 pandemic [1–4]. Several interventions have been put in place to prevent the spread of SARS-CoV-2 in hospitals in England including the use of enhanced personal-protective equipment (PPE), cohorting of suspected patients and symptomatic and regular asymptomatic testing of HCWs [5]. Testing policies in England for HCWs changed over time as testing capacity and technology increased, with the symptomatic PCR testing programme that was in place from the start of the pandemic expanded to include regular asymptomatic testing with lateral flow devices (LFDs) in November 2020. In August 2022 the asymptomatic testing programme was scaled back but symptomatic HCWs were still eligible for testing and then in April 2023 testing for symptomatic HCWs was also mostly halted [6, 7]. These constantly changing conditions (including also changes in the predominant SARS-CoV-2 variant, other non-pharmaceutical interventions and vaccine availability and status) make the effectiveness of a testing scheme at preventing infection difficult to assess from the data that have been collected during the pandemic. Therefore, in this work we conduct a simulation study to assess how various testing schemes would have performed in particular settings during the pandemic.

**Fig. 1:**
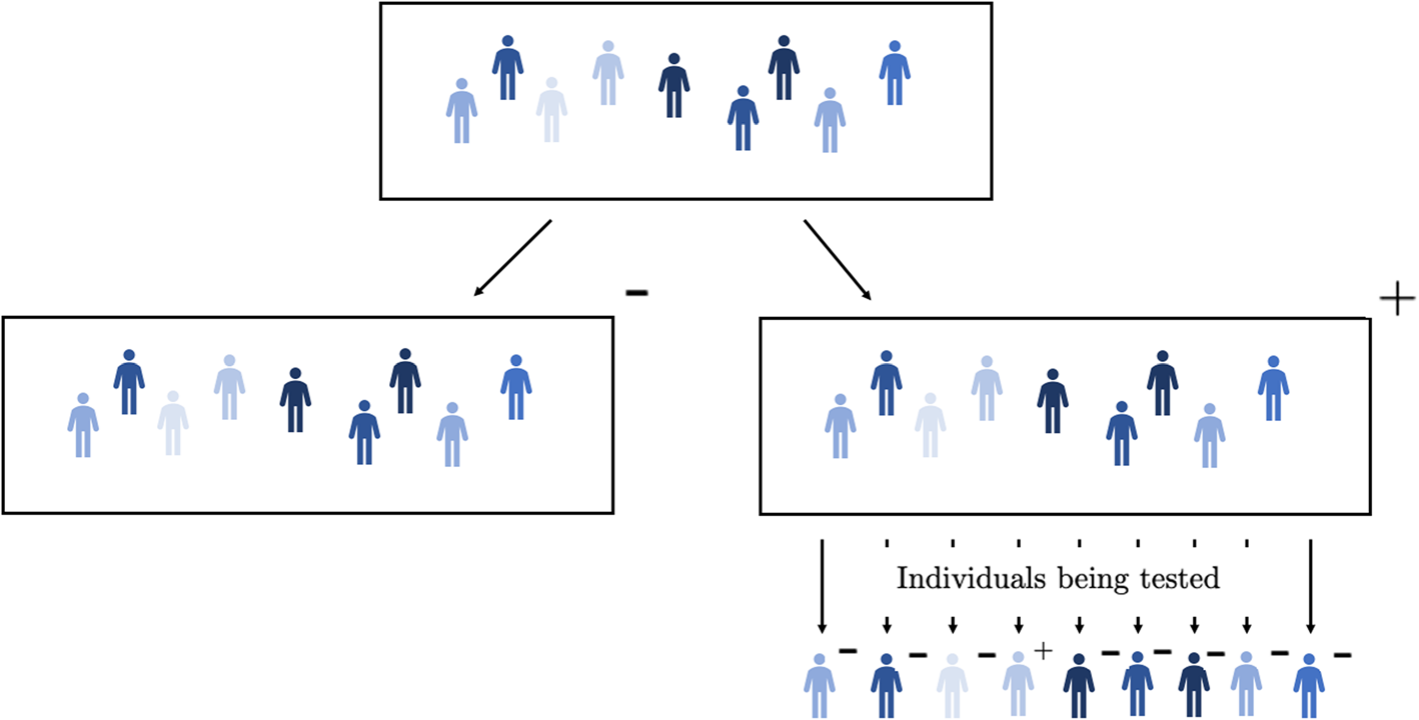
Diagram of the Dorfman Pooled testing design. This is for a pool of size 9. If the original pool was marked as negative then only one test would be used; if the pool was marked as positive then ten tests would be used. Figure taken from [10]

In pooled testing schemes, samples from multiple individuals are combined and analysed using a single test. In adaptive pooled testing, there are subsequent tests on groups or individuals if the combined sample comes back as positive. Pooled testing offers a way of introducing an asymptomatic testing scheme for HCWs that requires far fewer tests than an individual testing scheme. In this research, we will be using a Dorfman pooled testing design as shown in Fig. 1 [8], where individuals’ samples are combined into one pool of up to 12 individuals (the maximum pool size suggested by the NHS [9]) to be tested. If the pooled sample tests negative, then all individuals in the sample are marked as negative. However, if the sample tests positive then all individuals in the sample get tested individually.

Although researchers have recommended the use of pooled testing for key workers, there has never been any pooled testing of HCWs in hospitals in England [7, 11]. Nevertheless, pooled testing could be a tool to balance the cost of regular testing with the benefit of reduced HCW infections in hospital. It has the potential to be cost-efficient in terms of tests per infection prevented compared to a strategy where HCWs all test twice weekly, as we would expect many of the pooled HCW samples to be negative. As can seen be seen in Fig. 1, a pooled testing procedure can identify the same number of individuals as testing individually while using a lot fewer tests. In this work, we will explore whether pooled testing has the potential to make an asymptomatic testing scheme for HCWs more feasible during the height of the pandemic by reducing the number of tests required.

To evaluate the potential efficacy (in terms of reducing the overall and peak number of cases) and efficiency (the cost per individual tested) of pooled testing, we perform extensive comparative simulation studies using an individual-based model (IBM) of a ‘typical’ English hospital during various stages of the pandemic with a HCW population of 8000. These different stages include different community prevalence levels (such as low and high prevalence) of SARS-CoV-2 and the presence or absence of national lockdowns/social restrictions. Our comparison is between the baseline strategy of just testing symptomatic HCWs (as was done in English hospitals prior to April 2023) and the strategy of testing symptomatic HCWs combined with an additional pooled asymptomatic testing scheme for HCWs [6, 7]. In this paper, we produce the first assessment of the performance of pooled testing schemes for asymptomatic HCWs in hospitals and, more importantly for pandemic preparedness, we identify which factors should be considered when deciding whether pooled testing of HCWs should be implemented.

Additionally, we consider for the first time in the literature (as far as we are aware) a novel testing scheme that uses PCR tests for testing the pooled sample and then LFDs to test individuals from positive pools. This is in contrast to the standard procedure of using PCR tests for both the pool and the individual testing stages. We have proposed this new testing scheme because of the large reduction in both cost, laboratory resources and time of using LFDs.

## Methods

### Modelling of the hospital

We use a previously published IBM of nosocomial SARS-CoV-2 transmission [12, 13]. The model captures transmission of SARS-CoV-2 within and between patients and HCWs in a ‘typical’ English hospital. The model is calibrated to national patient admission and infection datasets from the SARS-CoV-2 immunity and reinfection evaluation (SIREN) study (HCW data), the NHSE Secondary Uses Service (SUS) dataset (patient data) and the NHSE situation report (Sit Rep) (for patient and HCW diagnosed infections and staff absence data) [14–16]. The hospital has 1000 beds and 8000 HCWs who work 12 hour shifts (around 50$\%$ on shift at any one time), and is split into multiple wards, with each ward containing a number of six-bed bays. The hospital bed occupancy rate is 85$\%$ at the start of the simulation and varies over time due to changes in COVID-19 admission rates and the stochastic nature of the length of stay for both infected and non-infected patients that are drawn from the SUS length of stay estimates for each new admission [14]. Patients can be susceptible or infected on admission to hospital, and those that are susceptible can be infected via transmission from other patients in the same bay or on the same ward, by HCWs while undergoing treatment, or by visitors at a rate determined by the community prevalence at that time (prevalence estimates obtained from [17]). While at work HCWs can be infected by other HCWs that are working on the same ward, HCWs anywhere in the hospital, patients they are treating, or in the community while off shift. Therefore, HCWs can either be infected in the hospital (nosocomially) or within the community. The simulations are calibrated to data on patient and HCWs infections from genomic studies [1, 18, 19]. HCWs are split into ward-based HCWs (around 30$\%$ of HCWs in the model), who only work on a single ward, and HCWs who work across the hospital. HCWs who work throughout the hospital visit 18 patients at random every shift to reflect data on the frequency of patient contacts for HCWs [20]. A full list of the parameter values used in the model is contained in Tables S1 and S2 in the Supplementary material and a more detailed description of the model is given by Evans et al. [21].

### Testing policies

As discussed previously in the introduction, the first stage of the testing policy combines individuals into one sample tested with a single PCR test. We model the PCR testing as taking two days: one day to collect the samples (either having them collected in one vial together or taken and send off separately and combined in a laboratory) and one day for the pooled PCR test. The second stage is individually testing HCWs in those pools that tested positive. If this re-testing is done with a PCR test then another day is needed for these tests; if an LFD is instead used, the test is done immediately to model LFDs results being available in less than 30 minutes [22]. It is assumed that in the follow-up individual tests, there is not the day delay for all the tests to be collected because individuals do not have to wait for others to also submit their sample and if they know the pooled test is positive, they are likely to want to know their individuals results. The cost of PCR and LFDs vary, with PCR test prices between £19 and £69 and LFD prices between £2 and £5 [23–25]. In this analysis, the cost of PCR tests was taken to be £31 and the cost of a LFD to be £4 [23, 24]. The LFDs and PCR tests also have different error profiles. We generate a CT value curve for each individual, allowing variation between individuals and by whether individuals are asymptomatic, or not as has been developed by Kissler et al. [26]. We model PCR tests as being able to detect asymptomatic and symptomatic cases after an incubation period with a probability of 1. LFDs are modelled as having an increasing probability of detecting cases as the CT values decrease, as described by Quilty et al. [27], using their reported threshold values on the CT values for detection. This difference in the follow-up test used will be referred to as *‘PCR and LFD’* for using LFD as the follow-up test and *‘PCR only’* for using a PCR test throughout the paper. If a HCW is in a positive pool, they continue working until they receive an individual positive test result, at which point they isolate for 7 days. In all other circumstances, the individuals will continue working normally.

The testing policies that are compared in this paper are referred to as *‘Baseline’*, *‘Pool Ward’* and *‘Pool Full’*. *Baseline* is where no asymptomatic testing is taking place and instead the only HCW tests being performed are individual PCR tests for symptomatic individuals. *Pool Ward* testing is where only ward-based HCWs are included into the pooled asymptomatic testing scheme and symptomatic HCWs are still being tested. In the ward-based testing approach, for each ward, the ward-based HCWs are put into the pool up to a maximum of 12 individuals in the pool. The other HCWs are put into the next pool up to a maximum of 12 individuals in that pool and so on. This means some pools may be quite small; however, due to the typical size of the ward (as the 8000 HCWs are split between 42 wards meaning on average each ward has 24 HCWs on it), most of the pools are of size 12. Ward-based HCWs from different wards will not be pooled and testing together and instead multiple small pools will be used. If one of the ward pools comes back positive then all HCWs on the corresponding ward will undergo follow-up testing, including those who were in a negative pool. The whole ward is tested (and not just those in the positive pool) because the infected HCWs could have infected more individuals on the ward since the original sample for the pools was taken. The final testing strategy, *Pool Full*, has both of the previous testing strategies (testing for symptomatic HCWs and pooled asymptomatic testing for ward-based HCWs) and has pooled asymptomatic testing for HCWs who are not ward-based. For testing the non-ward-based HCWs, these are again done in pools of up to 12 only containing non-ward-based HCWs, but in this case when a pooled test comes back positive then only those in the pool are tested individually.

### Simulation study

The key dimensions in the evaluations are: cost, HCW infections, number of HCWs isolating (as resulting staff shortages affect capacity to deal with pandemic consequences on healthcare, and can have other effects, such as on mortality) and patient infections. The impact on HCW infections is measured using the metric of expected percentage reduction in nosocomial infections of the testing policies over the baseline testing strategy. Only the nosocomial infections are considered as it is not possible to limit the infections that HCW workers get from the community using measures within hospitals. The patient infections are also assessed using the percentage reduction in patient infections over the baseline strategy. For both of these metrics, 95$\%$ prediction intervals are obtained across parameter sets and replications (as discussed later in this section) to assess if the values of the metrics are significantly different (statistically) from zero (i.e., that the pooled testing scheme used has no impact on the metrics versus the baseline). These inferences and significance calculations are based on the empirical distributions from the simulations and are not based on theoretical distributions. The metric for cost is the expected difference in economic cost in pounds calculated from the number of PCR tests and LFDs used between the baseline and the strategies involving pooled testing and the cost of PCR and LFDs. The metric for isolations is the total HCW isolating each day in the simulation for each of the testing methods. We do not weight these metrics to assign different levels of importance to them, as priorities may differ between policymakers and at various stages of the pandemic. For example, prior to mass-vaccination, the number of infections may be of more importance, due to increased risk of severe disease; whereas as we are transitioning back to ‘normal’ life, the number of isolations may be more important.

We will consider these metrics across various prevalence levels including low community prevalence (0.5–1%), medium community prevalence (1–2%), high community prevalence (2–4%) and very high community prevalence (4–8%) for a model duration of 100 days. These testing strategies have also been considered in the scenarios of *lockdown* and *not lockdown*. The results for not lockdown are what could be expected if the testing strategies were in use in a setting where community transmission is not restricted (like April 2023); while the values for *under lockdown* are relevant for responding to a future epidemic and retrospectively for a setting where community prevalence is restricted (like April 2020). Having a lockdown in place causes the relationship between the community prevalence and the admission rate of infected individuals to decrease by a factor of three. This means that in times of lockdown the proportion of HCW infections acquired in the community is lower than in the non-lockdown setting. The community prevalence data and admissions data used to model admission rates are taken from the Cambridge-PHE real-time transmission model estimates and SUS [14, 17, 28] respectively. The variants of SARS-CoV-2 considered in these scenarios respectively correspond to the variant circulating at the time, i.e. in the not lockdown setting the omicron variant is modelled, whereas wild-type is used for lockdown. Under the first lockdown there was a low community prevalence (end of March 2020 lockdown was declared) and in the later omicron setting (such as April 2023) there was a very high community prevalence [29–31]. Although these were the comparative prevalence levels at the times for COVID-19, this will not necessarily be the case in future epidemics. Therefore, we will evaluate the testing policy at all prevalence levels to explore the hypothetical situation of which testing scheme would have been preferable. In each of these settings, there are four testing policies to consider. These are *Pool Ward with PCR and LFDs*, *Pool Ward with PCR only*, *Pool Full with PCR and LFDs* and *Pool Full with PCR only*. These testing strategies are all compared to the baseline scenario of testing symptomatic HCWs individually. When describing the setting used, the prevalence level (i.e., low, medium, high or very high) will be noted, plus the strategy of which HCWs are tested (i.e., ward or full).

Each of the model settings are run for 20 parameters sets selected to represent a range of potential transmission scenarios and generated as described by Evans et al. [12]. These parameter sets vary in parameters that are unknown such as the probability of different groups in the hospital infecting each other. The parameter selection process used previously published work, where the initial parameter sets are generated by Latin hypercube sampling over a suitable range, [0,1] if the prior distribution is not known [32]. The outcome measures of patient and HCW infections over time from these parameter sets are then compared to national patient and HCW infection datasets (Sit Rep, SUS and SIREN [14–16]). The parameter ranges are then refined and new parameter samples are generated until the parameter sets generated represent both the patient and HCW infection level over time. Additional data such as the proportion of cases that are from patient-to-patient and patient-to-HCW contact are also incorporated from the literature and is varied between parameter bounds as included in the Supplementary material [1, 18, 19]. More details about the parameter selection procedure can be found in Evans et al. [32]. Each of these parameter sets are then replicated 20 times based on the number of replicates needed for the simulation output to stabilise (Fig. S1 contained in the Supplementary material). The means and prediction intervals were taken over the 400 model runs (over the 20 parameter sets each replicated 20 times to incorporate both parameter and stochastic uncertainty). The length of the simulation was kept to 100 days to keep the prevalence within the prevalence ranges for low, medium, high and very high prevalence.

## Results

### Additional cost

It is clear that having additional testing of HCWs will increase the overall cost of the testing policy by requiring a larger total number of tests. However, this cost increase is very different depending on the type of pooled testing in place and the type of tests used to implement it. Table 1 shows the expected additional total cost in pounds (averaged across the 20 parameters sets and 20 replications) and 95% prediction intervals for the testing policies over 100 days.

**Table 1:** The expected additional total cost in 1000 pounds (£) of the testing policies over the baseline testing strategy over 100 days not under lockdown (community transmission not restricted as in April 2023) for a HCW population of 8000, with 95$\%$ prediction intervals

	Low	Medium	High	Very High
PCR and LFD Full	375 $[$374, 377$]$	386 $[$385, 387$]$	396 $[$395, 397$]$	402 $[$401, 403$]$
PCR and LFD Ward	92.0 $[$91.5, 92.5$]$	94.3 $[$93.9, 94.6$]$	97.4 $[$97.1, 97.8$]$	99.3 $[$99.6, 98.9$]$
PCR only Full	1120 $[$1100, 1130$]$	1220 $[$1220, 1230$]$	1320 $[$1320, 1330$]$	1390 $[$1380, 1390$]$
PCR only Ward	258 $[$255, 261$]$	280 $[$279, 281$]$	317 $[$313, 320$]$	317 $[$315, 319$]$

Testing using LFDs as the follow-up test saves considerable additional cost. Indeed the cost of the *PCR and LFDs* policies for *Ward* is about 1/3 of using *PCR only* and about 1/4 for the *Full* testing strategy. However, it is important to note that *Pool Ward* testing using *only PCR* tests costs less than having a *Pool Full* testing policy using *PCR and LFDs*. The table of the additional cost of the testing schemes under lockdown (Table S5 contained in the Supplementary material) shows the same patterns as for not lockdown, but the cost of the testing policy is slightly cheaper, as more of the initial tests are negative.

### Reduction in HCW cases

A key aim of using this asymptomatic testing scheme is to reduce the infections in the HCW population. This metric is not only important because hospital policy should aim to reduce the risk of HCWs getting ill at work, but also because of the long-term repercussions of infections, such as long COVID [33]. Table 2 shows the average percentage decrease in nosocomial infections in HCWs from baseline for each of the testing methods not under lockdown. 95$\%$ prediction intervals have also been given and settings where the testing policy significantly reduces this percentage from the baseline are in bold.

**Table 2:** The expected percentage reduction in nosocomial infections in HCWs of the testing policies over the baseline testing strategy over 100 days not under lockdown (community transmission not restricted as in April 2023) for a HCW population of 8000, with 95$\%$ prediction intervals

	Low	Medium	High	Very High
PCR and LFD Full	**1.66** $[$**1.12, 2.21**$]$	**0.82** $[$**0.42, 1.23**$]$	**1.06** $[$**0.76, 1.35**$]$	**0.65** $[$**0.42, 0.89**$]$
PCR and LFD Ward	**1.86** $[$**1.29, 2.42**$]$	**1.33** $[$**0.95, 1.70**$]$	**1.54** $[$**1.25, 1.84**$]$	**0.90** $[$**0.68, 1.13**$]$
PCR only Full	**3.93** $[$**3.26, 4.60**$]$	**2.94** $[$**2.45, 3.42**$]$	**0.75** $[$**0.15, 1.35**$]$	-0.05 $[$-0.59, 0.50$]$
PCR only Ward	**4.31** $[$**3.55, 5.07**$]$	**3.02** $[$**2.55, 3.49**$]$	**1.03** $[$**0.40, 1.66**$]$	0.41 $[$-0.14, 0.95$]$

**Fig. 2:**
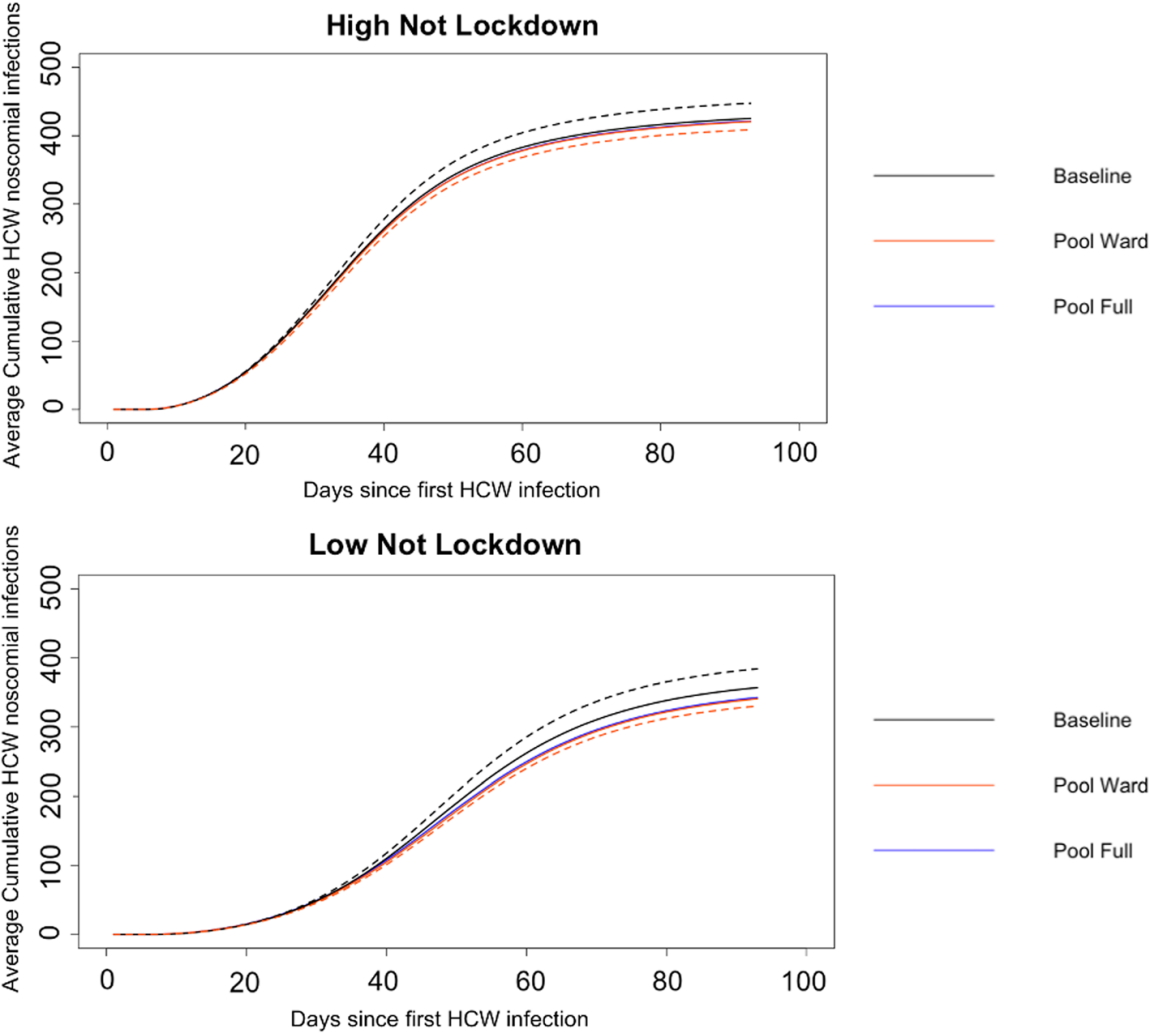
Cumulative average nosocomial infections across parameter sets and replications against time for a hospital with 8000 HCWs at low and high prevalence not during a lockdown (community transmission not restricted as in April 2023) for PCR only testing strategy. The black dotted lines denote the upper 95$\%$ prediction interval bound for the baseline; the orange dotted lines denote the lower 95 $\%$ prediction interval bound for the *Pool Ward* testing policy

For nearly all prevalence values, *PCR and LFD Ward*, *PCR only Full* and *PCR only Ward* testing strategies significantly decrease nosocomial infections. We can see the percentage decreases in nosocomial infections are modest, with about a 2$\%$ reduction in nosocomial HCW infections. This percentage decrease corresponds to a 165 decrease in total HCW infections for the case of low prevalence for *Pool Ward* testing and 127 decrease in the medium prevalence setting for *Pool Ward* testing using only PCR testing. The results for the total decrease in HCW infections are given in Table S6 (lockdown) and Table S7 (not lockdown) in the Supplementary material. The testing policies appear to be less effective for higher prevalence levels compared to lower ones in the not lockdown setting. This could be expected, as pooled testing works best for lower prevalence, where the initial pools are more likely to test negative. The difference in performance under low and high prevalence can be more easily seen by considering Fig. 2 showing the cumulative nosocomial infections, for high and low prevalence not during lockdown.

In Fig. 2, we see adding the asymptomatic pooled testing design slows down the rate of increasing COVID-19 infections. Although the difference in time when the number of infections reaches a certain level may not seem substantial, this gives the hospital more opportunity to prepare for low staffing levels (such as re-scheduling non-emergency appointments or bringing other HCWs into the hospital).

The percentage reduction in nosocomial infections and the conditions that significantly reduce this percentage are similar between lockdown and not lockdown. However, imposing lockdown improves the performance of the testing schemes at high and very high community prevalence. Additionally, PCR-only testing policies perform better than PCR and LFD even for high and very high prevalence when under lockdown. When looking at the cumulative infections, lockdown greatly reduces the total cumulative infections and the reduction in cumulative infections due to the testing strategies is more clearly seen (see Table S8 and Fig. S2 in the Supplementary materials).

In all of these settings, it is not clear cut whether a testing policy of using *PCR and LFDs* or *only PCR* tests is better at reducing nosocomial HCW infections, especially as the reduction changes depending on whether community prevalence is low/medium or high/very high. This is because it is not clear cut whether better test precision or reduced time to get results is the more important consideration. In most settings, however, we see that *Pool Ward* performs better to *Pool Full*.

Overall having an additional asymptomatic (pooled) testing policy in most settings significantly decreases the number of HCW nosocomial infections. However, this effect is increased by having a low or medium prevalence, being under lockdown and using a Pool Ward testing scheme.

### Increase in HCW isolations

**Fig. 3:**
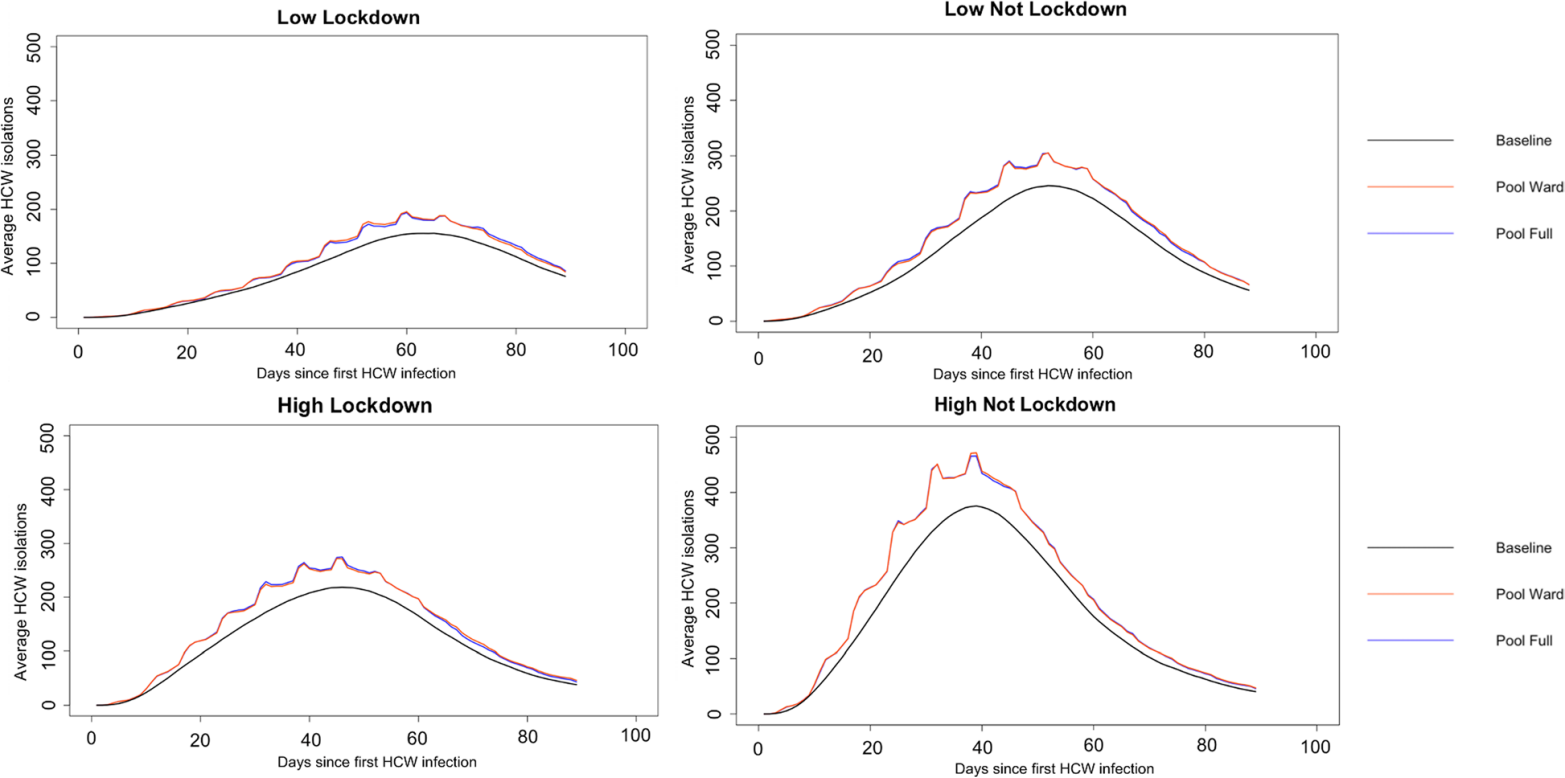
Average HCW isolations across parameter sets and replications against time for a hospital with 8000 HCWs at low and high prevalence in lockdown (community transmission restricted as in April 2020) and not lockdown (community transmission not restricted as in April 2023) settings. Low prevalence is on the top row and high prevalence is on the bottom row. Lockdown is the first column and not lockdown is the second

As would be expected, more HCW isolations occur as more infections are identified. In particular the peak of isolations was studied to highlight if there would be problems with staffing shortages. Across all pooled testing schemes, in both lockdown and not lockdown and across all prevalence settings, the peak of the HCW isolations was significantly higher, by around 20$\%$, than in the baseline case (see Table S11 for lockdown and Table S12 for not lockdown in the Supplementary material). Although it would be hoped that reducing HCW infections would reduce isolations, the impact of identifying more cases outweighs this. The graphs of the average isolations against time for low and high prevalence level and in the lockdown (community transmission restricted as in April 2020) and not lockdown setting (community transmission not restricted as in April 2023) are shown in Fig. 3. The lines for HCW isolations for the *Pool Ward* and *Pool Full* cases are less smooth because the pools for the wards are taken at the same time each week. This means just after the individual testing has been carried out, there are a lot of individuals going into isolation, therefore there is a jump in isolations for that period.

### Decrease in patient infections

As seen in the reduction in HCW cases section, adopting the additional pooled asymptomatic testing reduces the nosocomial HCW infections and it may be expected that by reducing HCW infections that this would reduce the number of nosocomial patient infections occurring. Table 3 shows the expected percentage reduction in patient infections for each of the pooling methods, for the various prevalence levels not under lockdown. Only those with a significant decrease in patient infections are in bold.

**Table 3:** Table of the expected percentage reduction in patient infections for the testing policies over the baseline testing strategy for 100 days for a HCW population of 8000 not under lockdown (community transmission restricted as in April 2023), with 95$\%$ prediction intervals

	Low	Medium	High	Very High
PCR and LFD Full	0.17 $[$-1.32, 1.66$]$	0.94 $[$-0.31, 2.20$]$	-0.02 $[$-1.06, 1.03$]$	-0.36 $[$-1.24, 0.52$]$
PCR and LFD Ward	-0.59 $[$-2.09, 0.90$]$	0.35 $[$-0.97, 1.67$]$	-0.56 $[$-1.60, 0.48$]$	-0.34 $[$-1.25, 0.56$]$
PCR only Full	-4.08 $[$-5.74, -2.41$]$	-1.40 $[$-2.78, -0.01$]$	-2.83 $[$-3.94, -1.71$]$	-1.80 $[$-2.84, -0.76$]$
PCR only Ward	0.22 $[$-1.21, 1.64$]$	**1.59** $[$**0.20, 2.98**$]$	0.71 $[$-0.35, 1.78$]$	0.78 $[$-0.16, 1.72$]$

There is no strong evidence that pooled testing for HCWs would reduce the patient nosocomial infections significantly. This is because the testing is only being carried out on HCWs, so patient nosocomial infections will not be identified. Also, even though HCW infections are reduced, patients can still be infected from visitors to the hospital (who are not part of any testing scheme) and other patients (who are not part of the asymptomatic testing scheme). Therefore, the asymptomatic pooled testing scheme is unlikely to impact patient infections so it may not be the best metric to judge on the scheme’s overall value for pandemic preparedness and management.

### Compromise between the metrics

When a policymaker is making a decision on whether to use an asymptomatic testing policy and, if so, which one to use, there are at least four metrics that should be considered: HCW infections, HCW isolations, cost of the testing scheme and patient infections (although patient infections are not considered here, as the asymptomatic pooled testing scheme has limited impact for all scenarios as demonstrated previously).

In these star plots, the ‘better’ a result is for a specific metric, the further to the outside the point is, i.e., further to the outside indicates a greater reduction in the number of HCWs infected, lower cost and a lower peak in the number of HCW isolations. To aid easier comparison between the star plots, they are all centred on the same grid coordinates. For the decrease in HCW infections, the minimum point (i.e., the centre point) is 0 and the maximum is 200. For peak isolations, the minimum is $-200$ (representing 200 more HCWs isolating at the peak compared to the Baseline testing strategy) and the maximum is 0. The cost in £1000s varies between –1400 and –90.

**Fig. 4:**
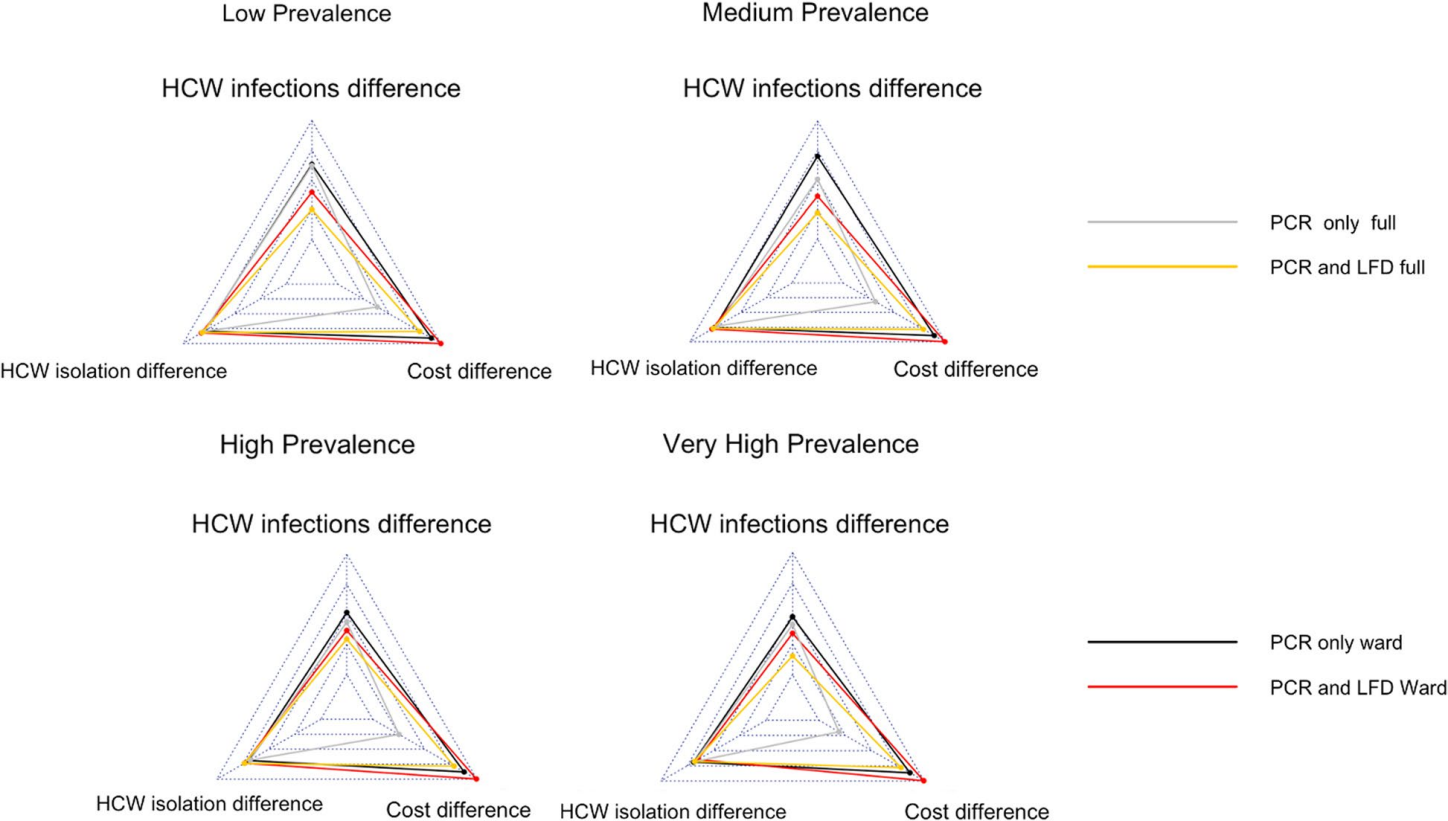
Star plot for four prevalence settings comparing *Pool Ward* with an LFD follow-up test, *Pool Ward* with all PCR tests, *Pool Full* with an LFD follow-up test and *Pool Full* with all PCR tests under lockdown (community transmission restricted like April 2020)

**Fig. 5:**
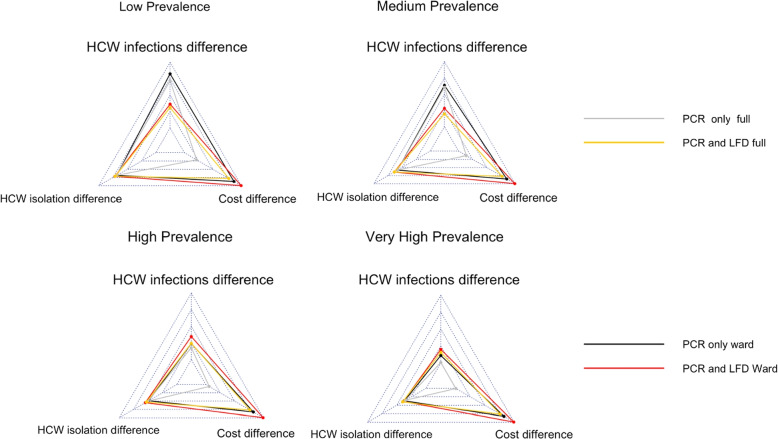
Star plot for four prevalence settings comparing *Pool Ward* with an LFD follow-up test, *Pool Ward* with all PCR tests, *Pool Full* with an LFD follow-up test and *Pool Full* with all PCR tests not under lockdown (community transmission restricted like April 2023)

Figure 4 is for the case of lockdown (community transmission restricted like April 2020) and Fig. 5 is for the case of not lockdown (community transmission is not restricted like April 2023).

We see that for the three metrics considered that *Pool Ward* appears to outperform *Pool Full*. Therefore, when we are considering testing policy, we should consider a *Pool Ward* scheme before a *Pool Full* one, especially if a key metric is cost of the testing scheme. The comparison is not so clear with *PCR and LFD* and *only PCR*, where instead policymakers would need to decide which of the metrics takes priority, that of cost difference (where PCR and LFD should be used) or that of HCW infections (where PCR only should be used). The exception to this scenario is in the high and very high community prevalence for the case of not lockdown where PCR and LFD performs very similarly, if not better, than only PCR in terms of decreasing HCW infections. Therefore, this suggests in the case of not lockdown and high or very high community prevalence, PCR and LFDs should be used, while in other situations it will depend on the metric of most importance to policymakers.

## Discussion

We have performed a simulation study using an IBM to evaluate the potential for an asymptomatic pooled testing scheme for HCWs in a hospital setting. We considered the situations of lockdown (community transmission restricted as in April 2020) and not lockdown (community transmission not restricted as in April 2023) to evaluate whether the usefulness of the testing scheme depends on whether we are at the early stages of a pandemic or later stages, when transitioning back to ‘normal life’. We also considered the various prevalence levels in each of these stages. This paper illustrates the potential of a pooled asymptomatic testing scheme and factors to be taken into account, highlighting a framework based on a realistic pathogen model, simulation of testing scheme and metrics of interest that can be used to evaluate testing policies in a future pandemic.

We found that there was a modest (albeit statistically significant) reduction in nosocomial HCW infections (around 2$\%$) when incorporating the asymptomatic pooled testing design into the hospital testing policy. This would suggest that policymakers should consider such a design if reducing HCW infections is vitally important, but they should not expect large reductions.

A novel pooled testing scheme was also considered where an LFD or a PCR test would be used for the follow-up testing. This comparison helps explore whether it is more important to have a considerably less costly test result back quickly or a more accurate (and more costly) test result. It was seen that in terms of reducing HCW infections that using only PCR tests performs better in most settings aside from high or very high community prevalence without a lockdown in place. *PCR and LFDs* had the considerable advantage of being 1/3-1/4 of the expected cost of *only PCRs*.

Interestingly, in most scenarios, the asymptomatic pooled testing scheme has the most impact under lockdown. This may indicate that a pre-prepared protocol should be in place for a future pandemic where first pooled testing for asymptomatic HCWs is implemented, then quickly paired with increased public health and social measures to improve the performance of the asymptomatic testing scheme. This protocol could be a part of the hibernation pandemic studies and interventions that are in place in the UK [34]. It also indicates that if the prevalence level becomes very high, it may be best to pause the asymptomatic pooled testing scheme or switch to using LFDs as follow-up testing as the benefit of having it in place is reduced.

Although there may be concerns about the feasibility and resulting error levels of a pooled testing scheme (when compared to an individual one), these concerns do not immediately rule out the value of pooled testing schemes as an effective intervention for policymakers. Pooled testing was implemented at the Saarland University Hospitals for asymptomatic patients and medical staff with pools of up to 30 individuals (with analysis of pool size being conducted by Lohse et al. [35]) where due to its success the pooled testing scheme was expanded to include residents and staff at nursing and care homes in Saarland [36, 37]. An asymptomatic testing scheme that included HCWs was also implemented by Fundación Biomédica Galicia Sur with pools of up to 20 individuals [36–38]. Pooled testing has also been considered in other settings during the pandemic such as in universities and long-term care facilities [36, 39]. The University of Cambridge testing scheme grouped individuals in the same household into pools up to a pool size of 10. The pooled testing scheme successfully identified individuals (for example, on the week of the 17th of January 2022, 16 infected individuals were identified from 15 positive pools [40]) and enabled estimates of the proportion of asymptomatic infections in the student community.

Although increased errors caused by dilution were not considered in our model for pools (they can easily be added when using this modelling in a future epidemic), we used the NHS’ recommended pool size where in their work with the University of Birmingham they found 100$\%$ concordance between the pools and individual samples i.e., all pools that contained a positive sample (as identified with individual PCRs) tested positive (seen in correspondence with Dr Andrew Beggs, University of Birmingham) [9]. Using pools with more individuals in them would decrease the cost of the testing schemes however this would need to be balanced by policymakers with the potential increase in false negatives caused by the dilution effect. Further research needs to be conducted to assess the dilution effect on larger pool sizes especially at what pool size the probability of false negatives is increased due to dilution of the positive samples. Additionally, if the testing for the pooled sample was taking place in a high throughout laboratory setting and the combining of samples was not possible in the laboratory, all members of a pool could instead be asked to put their sample into a single vial. This would however require more organisation in the hospital.

The key contribution of our work was evaluating an asymptomatic pooled testing scheme for HCWs in the hospital setting for COVID-19 considering different prevalence levels and amounts of community transmission. A novel pooled testing scheme involving LFDs, rather than PCR tests, in the follow-up was also both introduced and evaluated. Although the simulation framework present here currently uses the dynamics and properties of CoV-SARS-2, the simulation framework can be used in future pandemics by simply adjusting these parameters in the IBM. This would allow researchers to quickly assess the potential value (in terms of different metrics, for example nosocomial infections prevented) of an asymptomatic pooled testing scheme during the early stages of a pandemic. In the early stages of an epidemic, there may not yet exist a quick, less accurate, form of testing yet (like an LFD) so instead the analysis may consider only the relative benefits of the *Baseline* and *Ward PCR-only* schemes.

One limitation in our simulations is that the consequence of many HCWs being infected or isolating at the same time is not considered. For example, we did not consider a ‘tipping point’ where if over a certain percentage of HCWs are infected or isolating then the hospital ceases to be able to run effectively and so increases mortality rates in patients. Also, the effect of HCWs who are infected taking time off work even if they are not identified in a testing scheme is not included in the numbers who are isolating. Additionally, the impact of HCWs infecting their families and so needing to take time off work to care for them or the impact of HCWs having long COVID and so taking more absence in the future have not been considered. Also, this simulation only runs for 100 days so a greater reduction in cases would be expected if a larger time period was considered. We did not include these features in the model as there is no data on the proportion of individuals that this would affect, the effect this would have and when such a tipping point would be, therefore if this was included in the analysis the values taken would be arbitrary. However, by not including these features it makes our estimates of the positive impact of the pooled testing scheme conservative compared to what the true value could be. Therefore, the use of pooled testing policy in practice would need to consider these additional concerns.

In the proposed testing schemes, HCWs do not isolate when they are in positive pools. The impact of the testing scheme in terms of reducing HCW infections may be improved by having all HCWs isolate for the day until their individual test results comes back. Pooled testing with this design has seen to have a greater impact in the community setting than the pooled testing scheme proposed here [10]. However, this would need to be balanced against the increase in isolations. Additionally, the proposed asymptomatic testing policy is only for HCWs. As has been highlighted previously, HCWs and patients are infected by other patient and visitors who are not involved in the testing scheme. Asymptomatic testing schemes for HCWs and patients have been considered in long-term care facilities by Smith et al.; however, further research would be required to see if an asymptomatic scheme for patients or a testing scheme visitors would be beneficial enough in the hospital setting to justify the additional tests that would be needed [39].

A concern may be the accuracy of using LFDs, as it has been reported that accuracy of LFDs is lower in asymptomatic individuals versus symptomatic individuals [41]. However, this could be mitigated against by individuals who are in positive pools taking LFDs on subsequent days following the pool being positive rather than a single LFD at the time of the pooled result (from correspondence with Dr Tom Fowler, UKHSA). One positive LFD would mean that person would isolate and not need to take any more tests. The cost of such a testing scheme would be slightly higher than taking only one LFD but the cost would still be much lower than a PCR test.

The proposed testing schemes assume that only individuals in the same ward being able to be put into the pool together. This meant for certain wards, as the maximum pool size was 12, there were some pools with only a few individuals. Individuals were only pooled with others from the same ward because if the pool tested positive the whole ward would be tested rather than just those in the pool. The rationale behind this is because the infection was very likely to pass to others on the ward from an infected HCW. However, further analysis could go into exploring whether this is the most efficient way to pool so the least number of tests are used to find the most cases. For example, it may be beneficial to combine tests from different wards into pools and if this is positive then test all individuals in the wards that went into the pooled test. Alternatively, there may not be that much spread in a ward and it may be beneficial to just test individually those who were in the positive pools. This should be considered separately for the *LFD and PCR* and *PCR only* testing schemes as their different error profiles and length of time might make different schemes preferential.

Additionally, the impact of a testing policy depends greatly on the proportion of infections that are nosocomial in nature. For example, a *PCR only ward* testing policy reduces the number of HCW infections by 228 in the low community prevalence setting for not lockdown for the parameter set with the most nosocomial transmission (parameter set 9) and only by 26 for the parameter set with the least nosocomial transmission (parameter set 1). Therefore, the impact of the asymptomatic testing scheme will greatly depend on amount of nosocomial transmission. This means it could make sense to have different policies depending on the transmission dynamics in the hospital. However, this raises the further question on how to monitor and estimate this proportion. This is a developing area of research on understanding how genomic and epidemic data can be combined to estimate whether infections were nosocomically acquired [42]. Currently, however, although in the model we can distinguish whether a HCW was infected nosocomially or not, in reality this is not yet the case. Therefore, our testing policy needs to aim to identify infected individuals as soon as possible irrespective of where the infection was acquired.

## Conclusions

In this paper, we have shown ways in which pooled asymptomatic testing could feasibly be used alongside the symptomatic testing of HCWs and quantified some metrics of its expected performance. Whether individual policymakers want to implement HCW asymptomatic pooled testing depends greatly on the metrics of concern for the policymaker and the context (such as the pathogen, public health and social measures, variants and capacity of tests). If cost is a major objection, then an asymptomatic testing scheme may not be optimal, although a pooled asymptomatic scheme is more feasible than an individual testing one. If a compromise between the metrics is desired then the best policy to be put in place depends on the prevalence of the pathogen, whether public health and social measures are in place and the amount of nosocomial transmission in the hospital. In most scenarios, a *Pool Ward* testing design using only PCR performs better than *Pool Ward* with PCR and LFDs for reducing HCW infections and a *Pool Ward* strategy using PCR and LFDs is better in terms of cost. However, there are some scenarios (not lockdown and high or very high prevalence) when a Pool Ward design using PCR and LFDs should also be considered. Therefore, in the recent setting (April 2023) where no lockdown is in place and a very high prevalence rate would be expected (even if there are no population testing schemes in place to confirm this), Pool Ward with PCR and LFDs appears to be the preferable testing solution.

### Supplementary Information

**Additional file 1.**

## Data Availability

The data and the main code for this paper is available at https://github.com/beth-heath/EvaluatingHCW with the code from Evans et al available upon request [32]. The data that support the findings of this study is available from NHS Digital and UKHSA but restrictions apply to the availability of these data, which were used under license for the current study, and so are not publicly available.

## Abbreviations

HCWs: Healthcare workers
LFDs: Lateral flow devices
PCR: Polymerase chain reaction
PPE: Personal-protective equipment
IBM: Individual-based model
SIREN: SARS-CoV-2 immunity and reinfection evaluation
SUS: Secondary Uses Service
Sit Rep: NHSE situation report
