# Supplementary material for: Evaluating pooled testing for asymptomatic screening of healthcare workers in hospitals
Source: BMC Infect Dis. 2023 Dec 21;23:900. doi: 10.1186/s12879-023-08881-x (PMC10740241; doi:10.1186/s12879-023-08881-x)
Supplement: Supplementary file 1 — Additional file 1. [file 12879_2023_8881_MOESM1_ESM.pdf]

Table S1: List of parameter values used in our modelling taken from Evans et al, 2021

Proportion of occupied beds in trust	0.85
Number of HCWs	8000
Number of patients initially in the trust	860
Shape variable for Gamma distribution for incubation period	13.3
Rate variable for Gamma distribution for incubation period	4.16
Mean for log-normal distribution for onset period	1.434065
Standard deviation for log-normal distribution for onset period	0.6612
Number of wards	42
Number of bays	4
Number of beds in a shared room	6
Probability of patients being male	0.576
Shape component of Gamma distribution of age distribution	4.145
Scale component of Gamma distribution of age distribution	75.48
Shape parameter of LOS distribution Weibell distribution for susceptible patients	-0.1780082
Intercept for scale parameter for LOS distribution Weibell distribution for susceptible patients	2.331949
Age component of scale parameter for LOS distribution Weibull distribution for susceptible patients	-0.00546131
Female gender component of scale parameter for LOS distribution Weibull distribution for susceptible patients	-1.242609
Interaction of age and female gender component of scale parameter for LOS distribution Weibull distribution for susceptible patients	0.01424664
Shape parameter for LOS distribution Weibull distribution for symptomatically infected patients	0.07852536
Intercept for scale parameter for LOS distribution LOS distribution Weibull distribution for symptomatically infected patients	2.595361
Age component of scale parameter for LOS distribution LOS distribution Weibull distribution for susceptible patients	0.002301093
Female gender component of scale parameter for LOS distribution Weibull distribution for susceptible patients	-0.5465226
Interaction of age and female gender component of scale parameter for LOS distribution Weibull distribution for susceptible patients	0.00697802
Probability a patient infection is asymptomatic	0.4
Transmission rate to other patients	Varied between [0.028, 0.086] in the parameter sets
Transmission rate from HCW to patients	Varied between [0.00001, 0.00063] in the parameter sets
Indirect transmission rate from patients to other patients	Varied between [0.000013, 0.00069] in the parameter sets
Intercept of linear model for probability of death on discharge for infected patients	-0.00043572
X component of linear model for probability of death on discharge for infected patients	-0.00159802
X^2 component of linear model for probability of death on discharge for infected patients	7.30529×10^{-5}
Intercept of linear model for probability of death on discharge for non-symptomatic/uninfected patients	-7.96613
X component of linear model for probabilty of death on discharge for non-symptomatic/uninfected patients	0.00102
X^2 component of linear model for probability of death on discharge for non-symptomatic/uninfected patients	0.000896
Probability a symptomatic infected patient will be tested on admission	0.95
Probability non-infected patient will be tested at random on admission	0.05
Probability a symptomatically infected patient infected nosocomially will be tested per timestep	0.1
Timesteps from test to result	8
Probability a patient that develops symptoms after discharge will be readmitted within 14 days	0.2
Probability a susceptible patient will be exposed in admission (to be multiplied by the number of cases admitted that are known symptomatic)	0.002631579
Probability a patient will be retested after day 5 (per timestep)	0
Number of unique patients seen by a HCW every day (used to calculate exposure risk to COVID+ patients)	20
Day to start testing all admissions	0

Table S2: Continued list of parameter values used in our modelling taken from Evans et al, 2021

Day to start retesting on day 3	0
Day to start retesting on day 5	0
Probability of a non-infected patient will have COVID-19 like symptoms on admission	0.1
Proportion of new admissions that have previously recovered from COVID-19	0.5
Proportion of new admission that have previously recovered from omicron	0.05
Length of shift in hours (converted to timesteop within model code)	12
Probability a HCW that becomes infected is asymptomatic	0.4
Transmission probability from patients to HCWs per time step	0.0000025
Transmission probability from HCWs to other HCWs per timestep	Varied between [0.00001, 0.0007] in the parameter sets
Indirect transmission from HCWs to other HCWs	Varied between [0.0000001, 0.000153] in the parameter sets
Probability a HCW will self-isolate per timestep	0.01
Periodicity a HCW LFD testing (days)	3.5
Proportion of HCWs adhering to LFD testing regime	0.7
Number of days to be off work following a positive test	7
Day that LFD testing of HCW began	253
Scale of community acquisation rate for HCWs	Varied between [0.11, 0.67] in the parameter sets
Number of HCWs seen by a patient per day (for scaling transmission risk)	18
Probability a HCW is based on a ward instead of moving around the hospital	0.25
Day after vaccination that protection begins	21
Rate of vaccine wanning per time step	0.003
Efficacy of one vaccine dose	0.7
Efficacy of two vaccine doses	0.8
Efficacy of one vaccine doses against omicron	0
Efficacy of two vaccine doses against omicron	0.32
Efficacy of three vaccine doses against omicron	0.62
Efficacy of protection against omicron in those that have prior infection	0.44
Efficacy of one vaccine doses agInst omicron in those that have prior infection	0.44
Efficacy of two vaccine doses against omicron in those that have prior infection	0.6
Efficacy of three vaccine doses against omicron in those that have prior infection in those that have prior infection	0.71
Day that vaccination program in HCWs commences	281
Probability a patient in from a care home	0.02
Scaling factor for admission rate	1
Scaling factor for transmissibility from vaccinated individual	0.5
Scaling factor for transmissibility to individual vaccinated with one dose (trans=1-value)	0.7
Scaling factor for transmissibility to individual vaccinated with two doses (trans=1-value)	0.7
Scaling factor for transmissibility to individuals vaccinated with two doses (trans=1-value)	0.7
Scaling factor of transmission parameter to individual vaccinated with two doses (trans=1-value)	1.56
Scaling factor of transmission parameter for alpha variant (compared to wild-type)	1.56
Scaling factor of transmission parameter for delta variant (compared to wild-type)	1.99
Scaling factor of transmission parameter for omicron variant (compared to wild-type)	2.32

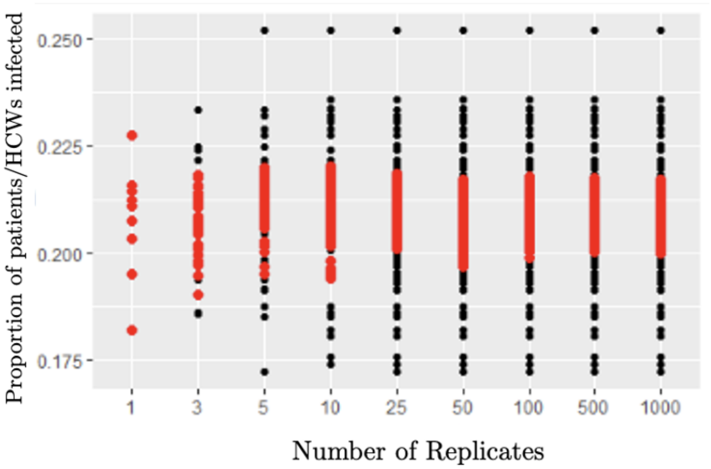

Figure S1: Figure of the proportion of patients infections/HCWs infections considered for a number of replicates ranging from 1 to 1000. The red points represents the mean of the number of runs given on the x-axis repeated 10 times.

Table S3: Table of tests used for all testing policies for 100 days not under lockdown for a HCW population of 8000. PCR and LFD denotes follow-up testing was done with an LFD and PCR only means follow-up testing was done with a PCR test. The number first written is the number of PCR tests and the number written in brackets is the number of LFDs.

	Low	Medium	High	Very High
Baseline (PCR and LFD Full)	201 (0)<br>[192 (0), 211 (0)]	250 (0)<br>[239 (0), 261 (0)]	282 (0)<br>[271 (0), 294 (0)]	331 (0)<br>[318 (0), 345 (0)]
PCR and LFD Full	8460 (29900)<br>[8450 (29500), 8460 (30300)	8470 (32700)<br>[8460 (32500), 8480 (33000)]	8500 (35300)<br>[8490 (35100), 8510 (35600)]	8540 (36900)<br>[8530 (36600) , 8550 (37200)]
PCR and LFD Ward	2280 (6920)<br>[2260 (6820), 2290 (7010)]	2320 (7510)<br>[2310 (7450), 2340 (7570)]	2370 (8160)<br>[2360 (8110), 2390 (8220)]	2430 (8530)<br>[2410 (8460), 2450 (8600)]
Baseline (PCR Full)	163<br>[157, 170]	196<br>[189, 202]	235<br>[228, 243]	316<br>[303, 330]
PCR only Full	36100<br>[35700, 36500]	39600<br>[39500, 39800]	43000<br>[42800, 43100]	45000<br>[44700, 45300]
PCR only Ward	8480<br>[8390, 8570]	9230<br>[9180, 9270]	9970<br>[9920, 10000]	10500<br>[10500, 10600]

Table S4: Table of tests used for all testing policies for 100 days under lockdown for a HCW population of 8000. PCR and LFD denotes follow-up testing was done with an LFD and PCR only means follow-up testing was done with a PCR test. The number first written is the number of PCR tests and the number written in brackets is the number of LFDs. The Baselines are slightly different between them due to statistical variation.

	Low	Medium	High	Very High
Baseline (PCR and LFD)	144 (0)<br>[133(0), 156 (0)]	167 (0)<br>[155 (0), 178 (0)]	196 (0)<br>[184 (0), 208 (0)]	231 (0)<br>[218 (0), 244 (0)]
PCR and LFD Full	8420 (22600)<br>[8400 (21700), 8430 (23500)]	8430 (25400)<br>[8420 (24700), 8440 (26200)]	8450 (28300)<br>[8440 (27700), 8470 (28800)	8470 (30900)<br>[8450 (30500) , 8480 (31200)]
PCR and LFD Ward	2210 (5250)<br>[2190 (5050), 2220 (5440)]	2230 (5890)<br>[2220 (5730), 2250 (6050)]	2260 (6500)<br>[2250 (6390), 2280 (6620)]	2300 (7060)<br>[2290 (6990), 2320 (7130)]
Baseline (PCR only)	130<br>[120, 141]	154<br>[143, 166]	183<br>[171, 195]	**217**<br>[**203, 231**]
PCR only Full	29000<br>[28200, 29900]	31900<br>[31100, 32600]	34700<br>[34200, 35300]	37400<br>[37100, 37700]
PCR only Ward	6870<br>[6680, 7060]	7520<br>[7350, 7680]	8190<br>[8070, 8310]	8790<br>[8720, 8860]

Table S5: The expected additional total cost in 1000 pounds (£) of the testing policies in over the baseline testing strategy for 100 days under lockdown (community transmission not restricted as in April 2023) for a HCW population of 8000.

	Low	Medium	High	Very High
PCR and LFD Full	357<br>[351, 363]	367<br>[363, 371]	374<br>[372, 376]	378<br>[376, 379]
PCR and LFD Ward	88.3<br>[86.4, 90.2]	90.9<br>[89.6, 92.3]	92.9<br>[92.0, 93.8]	94.9<br>[94.0, 95.7]
PCR only Full	896<br>[870, 923]	983<br>[960, 1010]	1070<br>[1050, 1090]	1150<br>[1140, 1160]
PCR only Ward	209<br>[203, 215]	228<br>[223, 233]	248<br>[245, 252]	266<br>[264, 268]

Table S6: Table of the total number of HCW infections reduced of the testing policies over the baseline testing strategy for 100 days under lockdown for a HCW population of 8000. PCR and LFD denotes follow-up testing was done with an LFD and PCR only means follow-up testing was done with a PCR test.

	Low	Medium	High	Very High
PCR and LFD Full	**50.2**<br>[**18.9, 81.5**]	**44.1**<br>[**21.3, 67.0**]	**58.9**<br>[**39.6, 78.3**]	**30.7**<br>[**10.4, 50.9**]
PCR and LFD Ward	**79.1**<br>[**47.0, 111**]	**72.1**<br>[**47.0, 97.1**]	**73.4**<br>[**54.8, 92.1**]	**68.0**<br>[**52.9, 83.1**]
PCR only Full	**124**<br>[**92.6, 155**]	**101**<br>[**76.8, 125**]	**87.8**<br>[**67.8, 108**]	**82.4**<br>[**65.3, 99.6**]
PCR only Ward	**126**<br>[**96.8, 156**]	**140**<br>[**114, 166**]	**103**<br>[**83.1, 123**]	**94.6**<br>[**78.0, 111**]

Table S7: Table of the total number of HCW infections reduced of the testing policies over the baseline testing strategy for 100 days not under lockdown for a HCW population of 8000. PCR and LFD denotes follow-up testing was done with an LFD and PCR only means follow-up testing was done with a PCR test.

	Low	Medium	High	Very High
PCR and LFD Full	**62.0**<br>[**44.0, 80.0**]	**39.2**<br>[**24.3, 54.2**]	**48.8**<br>[**36.7, 60.9**]	**30.8**<br>[**20.6, 40.9**]
PCR and LFD Ward	**72.6**<br>[**54.1, 91.1**]	**56.3**<br>[**42.3, 70.3**]	**69.1**<br>[**56.6, 81.7**]	**39.9**<br>[**30.2, 49.7**]
PCR only Full	**144**<br>[**123, 166**]	**121**<br>[**103, 140**]	**35.4**<br>[**10.9, 59.9**]	1.11<br>[-21.1, 23.3]
PCR only Ward	**165**<br>[**141, 189**]	**127**<br>[**109, 145**]	**48.2**<br>[**22.4, 74.1**]	21.7<br>[-0.75, 44.1]

Table S8: Table of the expected percentage reduction in nosocomial infections of the testing policies over the baseline testing strategy for 100 days under lockdown for a HCW population of 8000.

	Low	Medium	High	Very High
PCR and LFD Full	-0.64<br>[-2.62, 1.35]	0.69<br>[-0.37, 1.74]	**1.13**<br>[**0.46, 1.81**]	**0.85**<br>[**0.26, 1.45**]
PCR and LFD Ward	1.06<br>[-0.88, 3.00]	**1.59**<br>[**0.48, 2.69**]	**1.88**<br>[**1.24, 2.52**]	**1.84**<br>[**1.38, 2.30**]
PCR only Full	**3.22**<br>[**1.15, 5.30**]	**3.00**<br>[**1.87, 4.13**]	**2.37**<br>[**1.62, 3.12**]	**2.10**<br>[**1.58, 2.61**]
PCR only Ward	**3.37**<br>[**1.41, 5.33**]	**3.82**<br>[**2.51, 5.12**]	**2.83**<br>[**2.08, 3.57**]	**2.43**<br>[**1.90 2.95**]

Table S9: Table of the expected increase in peak HCW isolations for the testing policies over the baseline testing strategy for 100 days under lockdown for a HCW population of 8000. PCR and LFD denotes follow-up testing was done with an LFD and PCR only means follow-up testing was done with a PCR test.

	Low	Medium	High	Very High
PCR and LFD Full	36.7<br>[32.5,40.9]	45.5<br>[40.9, 50.0]	52.7<br>[48.5, 57.0]	64.1<br>[58.9, 69.4]
PCR and LFD Ward	35.4<br>[31.2, 39.6]	43.3<br>[38.7, 48.1]	53.5<br>[49.1, 57.8]	70.3<br>[65.8, 74.7]
PCR only Full	40.6<br>[35.3, 45.8]	53.5<br>[47.6, 59.3]	63.7<br>[58.7, 68.8]	86.5<br>[81.2, 91.7]
PCR only Ward	41.4<br>[36.3, 46.5]	48.5<br>[43.2, 53.7]	62.1<br>[57.3, 66.9]	89.4<br>[84.3, 94.5]

Table S10: Table of the expected increase in peak HCW isolations for the testing policies over the baseline testing strategy for 100 days not under lockdown for a HCW population of 8000. PCR and LFD denotes follow-up testing was done with an LFD and PCR only means follow-up testing was done with a PCR test.

	Low	Medium	High	Very High
PCR and LFD Full	57.3<br>[52.8, 61.8]	72.1<br>[68.0, 76.3]	94.3<br>[89.0, 98.6]	120<br>[115, 125]
PCR and LFD Ward	56.6<br>[52.2, 61.0]	72.1<br>[67.7, 76.6]	90.5<br>[85.9, 95.0]	122<br>[117, 127]
PCR only Full	63.2<br>[58.2, 68.2]	84.5<br>[79.7, 89.2]	99.3<br>[93.5, 105]	129<br>[122, 136]
PCR only Ward	62.9<br>[58.1, 67.7]	84.1<br>[79.6, 88.7]	102<br>[96.1, 108]	126<br>[119, 133]

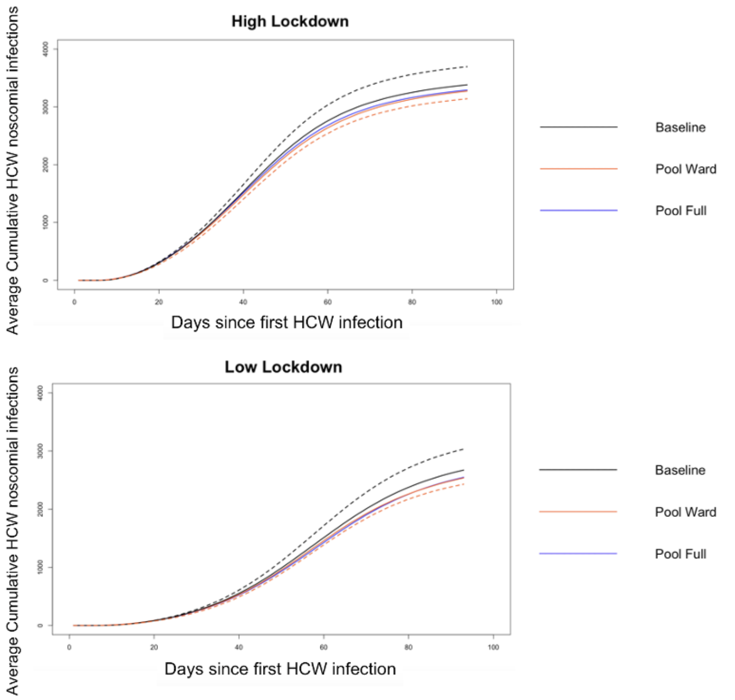

Figure S2: Cumulative average nosocomial infections across parameter sets and replications against time for a hospital with 8000 HCWs at low and high prevalence during a lockdown (community transmission not restricted as in April 2023) for PCR only testing strategy. The black dotted lines denote the upper 95% interval for the baseline; the orange dotted lines denote the lower 95 % interval for the pool ward case

Table S11: Table of the expected percentage increase in peak HCW isolations over Baseline policy for each testing policy for 100 days under lockdown for a HCW population of 8000. PCR and LFD denotes follow-up testing was done with an LFD and PCR only means follow-up testing was done with a PCR test.

	Low	Medium	High	Very High
PCR and LFD Full	20.4 [17.9, 23.0]	19.8 [18.0, 21.5]	21.0 [19.6, 22.4]	19.8 [18.4, 21.2]
PCR and LFD Ward	19.7 [17.2, 22.2]	18.9 [17.1, 20.7]	20.6 [19.2, 22.0]	21.3 [20.1, 22.5]
PCR only Full	21.1 [18.3, 23.9]	23.4 [21.2, 25.6]	24.3 [22.7, 25.9]	27.5 [26.0, 28.9]
PCR only Ward	20.7 [18.3, 23.0]	21.9 [19.8, 24.0]	24.1 [22.6, 25.6]	28.4 [27.0, 29.8]

Table S12: Table of the expected percentage increase in peak HCW isolations over Baseline policy for each testing policy for 100 days not under lockdown for a HCW population of 8000. PCR and LFD denotes follow-up testing was done with an LFD and PCR only means follow-up testing was done with a PCR test.

	Low	Medium	High	Very High
PCR and LFD Full	20.4<br>[19.1, 21.7]	22.8<br>[21.7, 23.9]	23.3<br>[22.4, 24.2]	23.4<br>[22.6, 24.2]
PCR and LFD Ward	20.5<br>[19.2, 21.8]	22.7<br>[21.5, 23.9]	22.5<br>[21.5, 23.4]	23.6<br>[22.8, 24.4]
PCR only Full	24.0<br>[22.5, 25.4]	27.0<br>[25.7, 28.3]	25.7<br>[24.2, 27.1]	26.1<br>[24.7, 27.6]
PCR only Ward	24.5<br>[23.0, 26.0]	27.1<br>[25.8, 28.3]	26.4<br>[25.0, 27.8]	25.5<br>[24.1, 27.0]

Table S13: Table of the expected percentage reduction in patient infections for the testing policies over the baseline testing strategy for 100 days for a HCW population of 8000 with a 95% prediction interval for under lockdown.

	Low	Medium	High	Very High
PCR and LFD Full	-6.60<br>[-11.1, -2.06]	-0.69<br>[-3.19, 1.82]	-0.92<br>[-3.80, 1.97]	-8.05<br>[-13.0, -3.10]
PCR and LFD Ward	-3.75<br>[-9.18, 1.69]	-0.85<br>[-3.58, 1.88]	-0.75<br>[-3.28, 1.77]	-1.04<br>[-3.09, 1.02]
PCR only Full	**3.14**<br>[**0.34, 5.94**]	-0.17<br>[-2.87, 2.53]	**2.36**<br>[**0.46, 4.26**]	0.49<br>[-1.32, 2.30]
PCR only Ward	2.11<br>[-0.58, 4.80]	0.22<br>[-2.45, 2.88]	**2.41**<br>[**0.45, 4.37**]	-0.85<br>[-2.72, 1.02]

Table S14: Table of the expected total reduction in patient infections for the testing policies over the baseline testing strategy for 100 days for a HCW population of 8000 with a 95% prediction interval for not under lockdown.

	Low	Medium	High	Very High
PCR and LFD Full	2.22<br>[-14.4, 18.8]	16.8<br>[-5.55, 39.0]	0.89<br>[-24.2, 25.9]	-20.5<br>[-45.1, 4.05]
PCR and LFD Ward	-4.81<br>[-21.6, 11.9]	6.29<br>[-16.0, 28.6]	-17.7<br>[-43.1, 7.72]	-17.1<br>[-41.9, 7.74]
PCR only Full	3.75<br>[-11.3, 18.8]	**23.5**<br>[**0.30, 46.7**]	4.72<br>[-20.1, 29.5]	-1.73<br>[-25.8, 22.4]
PCR only Ward	-4.77<br>[-20.1, 10.6]	**32.6**<br>[**7.60, 57.5**]	12.7<br>[-13.2, 38.5]	12.0<br>[-12.9, 36.8]

Table S15: Table of the expected total reduction in patient infections for the testing policies over the baseline testing strategy for 100 days for a HCW population of 8000 with a 95 % prediction interval for under lockdown.

	Low	Medium	High	Very High
PCR and LFD Full	-0.32<br>[-10.7, 10.1]	-2.51<br>[-14.2, 9.22]	-0.82<br>[-16.1, 14.5]	-33.2<br>[-55.7, -10.7]
PCR and LFD Ward	8.72<br>[-3.17, 20.6]	2.44<br>[-10.1, 15.0]	3.03<br>[-11.7, 17.8]	-8.24<br>[-26.1, 9.61]
PCR only Full	**16.5**<br>[**5.61, 27.4**]	4.44<br>[-8.31, 17.2]	12.0<br>[-2.74, 26.7]	-9.67<br>[-27.6, 8.28]
PCR only Ward	**11.5**<br>[**1.31, 21.7**]	4.37<br>[-8.34, 17.1]	11.1<br>[-3.75, 25.9]	-18.6<br>[-27.4, 0.21]

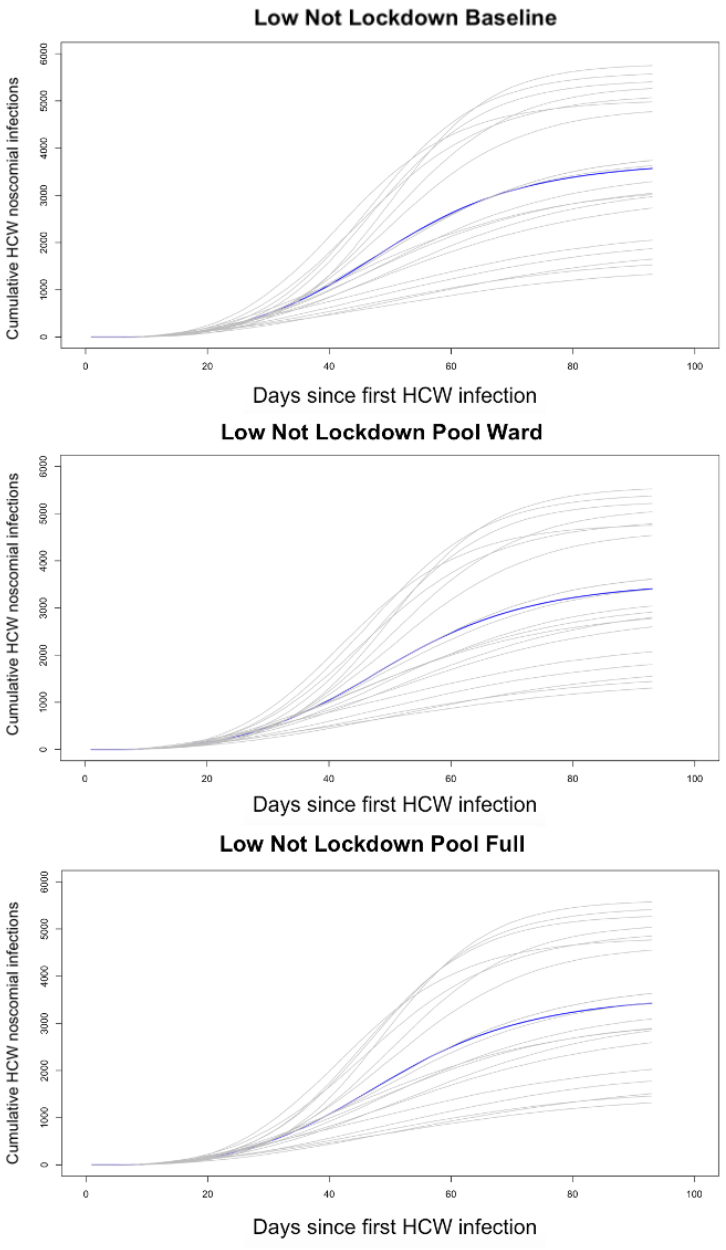

Figure S3: Cumulative graphs for HCW infections showing the 20 parameter sets (in grey) and the average of those parameter sets (in blue) for the three different testing methods for low prevalence not in a lockdown.

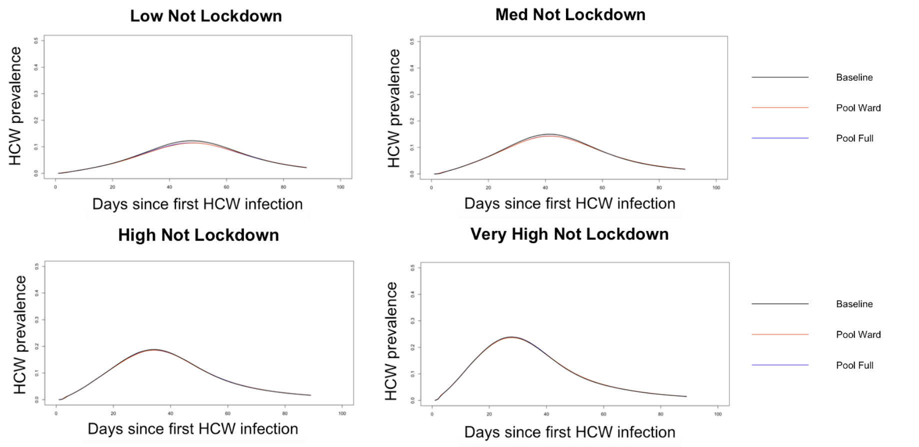

Figure S4: Prevalence in HCWs across the four community prevalence levels and three testing strategies for the non lockdown setting.
